# Hepatic Porphyria Presenting with Persistent Abdominal Pain: A Case Report and Literature Review

**DOI:** 10.30699/ijp.2025.2066613.3491

**Published:** 2025-11-11

**Authors:** Ying Yu, Lixia Yu, Minghui Li, Mengjie Ma, Yuwen Zhu, Jiacong Shen

**Affiliations:** 1 Shaoxing Joint Training Base Zhejiang Chinese Medical University, Hangzhou 310053, Zhejiang Province, China; 2 Department of Infectious Disease, Shaoxing People’s Hospital, 568 Zhongxing Road, Shaoxing 312000, China

**Keywords:** Hepatic porphyria, Abdominal pain, Genetic testing, Liver biopsy, Case report

## Abstract

**Background & Objective:**

Hepatic porphyria is an autosomal dominant disorder characterized by a deficiency in enzymes involved in hepatic porphyrin metabolism. Disruptions in this metabolic pathway can be precipitated by various factors, including physical exertion, psychological stress, fasting, infections, and drug withdrawal. Clinically, the condition manifests as episodic lower abdominal colic and a range of neuropsychiatric symptoms.

**Case Presentation:**

A 74-year-old male farmer presented with a four-month history of intermittent abdominal pain, abdominal distension, generalized weakness, and anorexia. The diagnosis of hepatic porphyria was established through a combination of imaging studies, laboratory investigations, liver biopsy, and genetic testing, which revealed a pathogenic c.587G>T (p.C196F) mutation in the FECH gene. The patient exhibited mild cutaneous lesions along with significant abdominal pain, abdominal distension, accompanied by constipation, nausea, and vomiting.

**Conclusion:**

This case highlights the diagnostic challenges and poor prognosis of hepatic porphyria when specific therapies are unavailable. Early recognition and genetic confirmation are vital for guiding management, and clinicians should suspect porphyria in patients with unexplained abdominal pain and liver dysfunction.

## Introduction

Porphyria refers to a group of rare metabolic disorders caused by defects in specific enzymes involved in the heme biosynthesis pathway. These disorders result in the accumulation of porphyrins or their precursors, leading to various clinical manifestations, primarily affecting the liver, nervous system, and occasionally the skin (1). Porphyrins are mainly synthesized in the bone marrow and liver (2). According to the site of porphyrin metabolic disorders, it can be divided into erythropoietic porphyria and hepatic porphyria (3, 4). There are three main clinical manifestations of hepatic porphyria: abdominal pain, photosensitive skin lesions, and neuropsychiatric abnormalities (5). Due to the low incidence of hepatic porphyria, complex and nonspecific clinical manifestations, it is easy to misdiagnose the case. Hepatic porphyria can be divided into four types (table 1): i) Acute intermittent porphyria (AIP), ii) Hereditary Coproporphyria (HCP); iii) Variegate Porphyria (VP); iv) ALA Dehydratase Deficiency Porphyria (ALADP) (6, 7).

**Table 1:** 

Type of Hepatic Porphyria	Enzyme Deficiency	Inheritance Pattern
Acute Intermittent Porphyria (AIP)	Porphobilinogen deaminase (HMBS)	Autosomal dominant
Hereditary Coproporphyria (HCP)	Coproporphyrinogen oxidase (CPOX)	Autosomal dominant
Variegate Porphyria (VP)	Protoporphyrinogen oxidase (PPOX)	Autosomal dominant
ALA Dehydratase Deficiency Porphyria (ALADP)	ALA dehydratase (ALAD)	Autosomal recessive (very rare)

The signs of hepatic porphyria can vary widely but the main signs like severe stomach pain, bloating, and bile buildup are quite uncommon (8). The aim of this case report is to describe the clinical, biochemical, histopathological, and genetic features of a patient with hepatic porphyria and to highlight the diagnostic challenges associated with this rare condition (9). Here, we report a diagnosed patient and analyze the patient's clinical manifestations and provide appropriate treatment as early as possible.

## Case Presentation

A 74-year-old male farmer was hospitalized for intermittent abdominal pain, fatigue, and loss of appetite lasting four months. The patient has a history of blisters, swelling, and tingling on the skin upon exposure to sunlight since childhood. The patient reported abdominal pain, abdominal distension, fatigue, and loss of appetite over the past four months, with no accompanying symptoms of nausea, vomiting, diarrhea, chills, or fever. The gastroscopy report indicated chronic atrophic gastritis with erosion, bile reflux, and an active gastric ulcer (A2), recommending follow-up after treatment, as summarized in the chart. The pathological report indicated severe chronic inflammation (active phase) and mild intestinal metaplasia in the gastric angle, accompanied by mild epithelial dysplasia in the local area, and suggested further examination post-treatment. The sigmoid colon exhibited a tubular adenoma accompanied by low-grade intramucosal carcinoma. The sigmoid colon exhibited a tubular adenoma with focal low-grade dysplastic changes approaching early intramucosal carcinoma. However, no invasion beyond the lamina propria was noted, and the lesion was entirely removed endoscopically. Given its intramucosal location and absence of high-risk features, the lesion holds minimal risk for metastasis. Following the identification of H. pylori (2+), the patient underwent a two-week course of anti-H. pylori therapy and did not return to the doctor. He was not experiencing diarrhea, but he frequently experienced yellow urine, skin and sclera jaundice, and sporadic stomach pain. For instance, the exposed skin would blister, swell, and sting after 20 to 60 minutes in the sun. It might even develop skin changes that resemble burns and sunburns. In most cases, the symptoms would go away in two to three days, although they would reappear after exposure. The skin on his face, hands, forearms, and lower legs had obvious pigmentation deposition, as well as multiple blisters, facial pigmentation deposition, and thickening changes, and several dysplastic atrophic scars with sizes of 3-6 mm on the back of his hands and forearms. His sibling did not exhibit comparable symptoms, while one of his sisters did. Three years later, he was diagnosed with vitiligo after the emergence of scattered white patches on his body. The patient was also diagnosed with a reversible inguinal hernia and did not receive any special treatment.

In the physical exam, the body temperature was 36.5℃, the pulse rate was 72 beats/minute, respiratory rate was 18 breaths/minute, and blood pressure was 105/61mmH Despite yellowing skin and sclera, the patient was awake and mentally well. Specific exam results were: Dark brown skin with white patches, yellowing, and keratosis on exposed parts and abdomen. The back of the hands had thick, cobblestone-like skin. Right anterior tibia coloration and local scabbing were present without bleeding or exudation. Liver palms and several spider nevi on the chest were observed and no wet or dry rales were heard in the coarse breath sounds. The heart beat regularly. A 5 x 4 cm mass was felt in the left inguinal region of the soft abdomen. There was modest lower abdominal pain without rebound. The liver and spleen were not palpable below the costal border. Costovertebral angle tenderness was absent, and dullness was elicited over the renal area, indicating positive abdominal shifting dullness. 2 mL of peripheral blood was collected from the patient for different testing as shown in table 2. 

Medical report containing various test results in table 2 and table S1 in supplementary file. ALT, AST, and bilirubin (Total, direct and indirect), albumin and GGT tests were assessed multiple times under different biochemical panels; results from both are presented here to reflect test variation during hospitalization.

**Table 2:** 

Category	Test/Observations	Result/Remarks	Normal Ranges
Blood Routine	White blood cell count	5.13 x10^9/L	3.5–9.5 x10^9/L
	Neutrophil percentage	82.2%	40.0–75.0%
	Hemoglobin	104 g/L	115–150 g/L (female), 130–175 g/L (male)
	Platelet count	67 x10^9/L	125–350 x10^9/L
	hs-CRP	9.68 mg/L	0–6 mg/L
	Reticulocyte percentage	4.67%	0.5–1.5%
Liver Function	Albumin Albumin	32.4 g/L 33.4 g/L	40–55 g/L ==
	ALT ALT ALT	57.6 U/L (i) 64 U/L (ii) 65.2 U/L (iii)	9–50 U/L == ==
	AST AST AST	88.1 U/L (i) 95.6 U/L (ii) 113.8 U/L	15–40 U/L == ==
	Total bilirubin Total bilirubin Total bilirubin	158.7 μmol/L (i) 199.7 μmol/L (ii) 198.6 μmol/L (iii)	5–21 μmol/L == ==
	Direct bilirubin Direct bilirubin Direct bilirubin	126.3 μmol/L (i) 100.4 μmol/L (ii) 103.5 μmol/L (iii)	0–3.4 μmol/L == ==
	Indirect bilirubin Indirect bilirubin Indirect bilirubin	32.4 μmol/L (i) 99.3 μmol/L (ii) 95.1 μmol/L (iii)	1.5–18 μmol/L == ==
	GGT GGT	158.8 U/L (i) 157.4 U/L (ii)	10–60 U/L
	Serum total bile acid	150.2 μmol/L	0–10 μmol/L
Urine Routine	Bilirubin	Positive	Negative
	Urobilinogen	Positive	Negative or weakly positive
Coagulation	Prothrombin time	14.2 sec	10–13.5 sec
	Prothrombin time ratio	1.23	0.85–1.20
	INR	1.19	0.85–1.15
ECG	Sinus bradycardia	51 bpm	60–100 bpm
Tumor Markers	CEA	5.3 ng/ml	0–5 ng/ml
	CA 50	56.3 u/ml	0–25 u/ml
	CA 125	74.22 u/ml	0–35 u/ml
	Pre-cancer antigen	Normal	0–13.4 ng/ml (AFP normal range)
Other Tests	Total protein	57.6 g/L	65–85 g/L
	Creatinine	45.6 μmol/L	59–104 μmol/L
	Uric acid	125.9 μmol/L	208–428 μmol/L (male), 155–357 μmol/L (female)
Iron Studies	Serum Total Iron Binding Capacity	21.8 µmol/L	45–75 µmol/L
	Unsaturated Iron Binding Capacity	3.1 µmol/L	31–51 µmol/L
	Transferrin Saturation	85.8%	20–55%
	Iron Level	5.33 mmol/L (Low)	9–32 µmol/L
Chest CT	Pulmonary Nodules	Multiple nodules; follow-up in 6 months	Not Applicable
	Emphysema	Both lungs	
	Pleural Thickening	Slight	
	Pericardial Effusion	Small amount	
	Mediastinal Lymph Nodes	Visible	
Abdominal Imaging	Ascites (Abdominal)	35mm (deepest)	Not Applicable
	Ascites (Liver-Kidney Fossae)	5mm	
	Liver Cysts	Multiple cysts	
	Gallbladder Wall Thickening	Wall thickening	
Ultrasound	Left Inguinal Hernia	Considered	Not Applicable
	Splenomegaly, Kidney Stone, Ascites	Small stone and ascites noted	
Heart Ultrasound	Heart Valves	Aortic, mitral, tricuspid, pulmonary	Not Applicable
	Valve Regurgitation	Mild regurgitation	
MRI	Liver Cysts	Multiple	Not Applicable
	Splenomegaly	Enlarged	
	Kidney Cysts	Small and complex cysts	
	Ascites	Present	

## Results

**Fig. 1:**
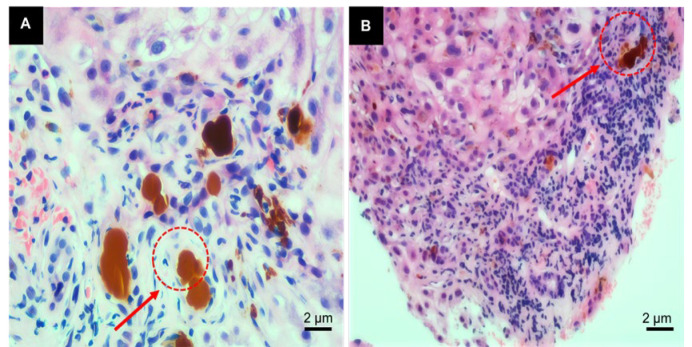
**A**) Dark brown protoporphyrin deposits are visible in hepatocytes, Kupffer cells, bile canaliculi, and bile duct lumens, indicating pigment accumulation in hepatic EPP. B) Portal inflammation, interface hepatitis, and ductular reaction are observed, reflecting chronic hepatic injury associated with EPP**.**

**Fig. 2:**
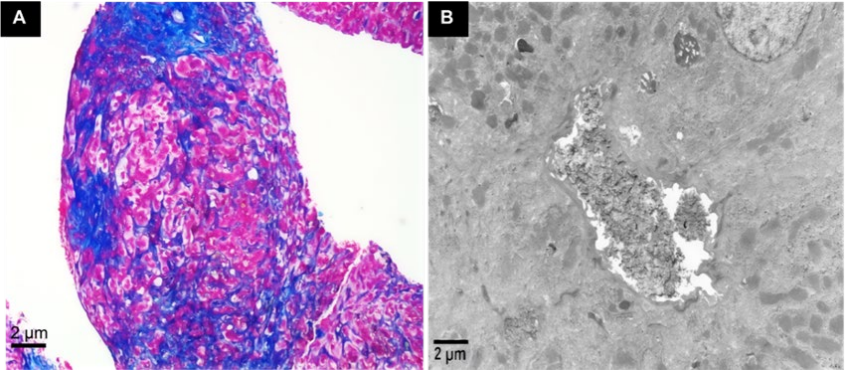
**A) Maltese Cross-shaped birefringent crystals are seen under polarized light, consistent with protoporphyrin crystallization. B)** CK7 and CK19 immunostaining show positive bile duct epithelium with minor ductular hyperplasia; approximately 70% of hepatocytes also exhibit CK7 positivity.

**Fig. 3:**
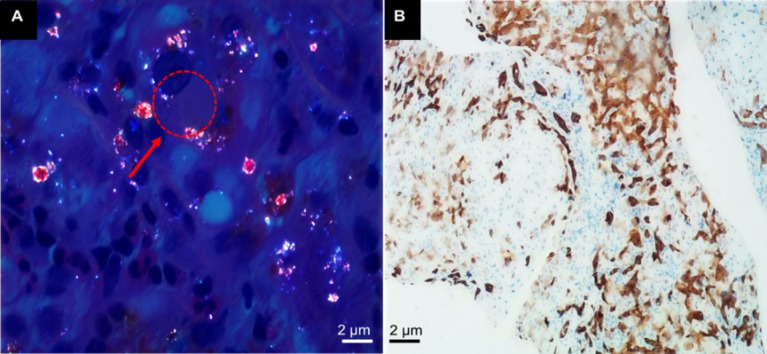
A) Masson and bitter almond-red staining reveal portal fibrous tissue hyperplasia, bridging and hair-like fibrosis, interlobular fibrous septa, cholestatic pigment in hepatocytes, bile duct dilation, crystallization, and loss of canalicular microvilli. B) Electron microscopy reveals cholestatic pigment accumulation in hepatocytes, dilated bile canaliculi, visible crystals, and loss of canalicular microvilli.

Liver biopsy showed varying sizes of portal areas with fibrous tissue proliferation and bridging fibrosis. In the portal areas (Fig 1.A), there was a moderate number of lymphocytes, some plasma cells, and neutrophils with mild to moderate interface inflammation, numerous focal necrosis, and pigment granules of bile stasis seen in some hepatocytes (Fig 1.B). Many small bile ducts and canaliculi were dilated with bile stasis, and brownish-red bile thrombi (visible as "Maltese cross" under polarized light microscopy) were observed (Fig 2.A). Immunohistochemistry showed CK7 and CK19-positive bile duct epithelium, mild bile duct proliferation, and about 70% CK7-positive hepatocytes (Fig 2. B). CD68 revealed a small number of activated Kupffer cells; hepatocytes were swollen with round nuclei, smooth nuclear membranes, and no endoplasmic reticulum dilation. The majority of mitochondria had normal appearance, while a few were swollen with irregular larger size but no cristae expansion. Masson and Picrosirius red staining showed fibrous tissue proliferation and bridging fibrosis in the portal areas (Fig 3.A). Hepatocytes contained varying amounts of pigment granules of bile stasis, and localized bile duct dilation and stasis with visible crystals, decreased or absent microvilli on luminal surface. Collagen fiber bundles were deposited between regional hepatocytes and sinusoids (Fig. 3B). Based on comprehensive light microscopy, immunohistochemistry, special staining, and electron microscopy, the findings were consistent with porphyria, suggesting a tendency toward the original porphyria-heme deposition porphyria. Moderate inflammation and fibrosis corresponded to the modified Scheuer score: G3S3.

### Genetic testing

**Fig 4:**
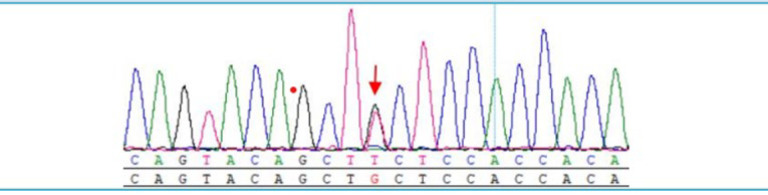
Sanger sequencing confirms a c.587G>T (p.C196F) mutation in the FECH gene, alongside identified mutations in TJP2 and UGT1A1

Whole-exome sequencing revealed mutations in the FECH gene c.587G>T (p.C196F, Fig.4), the TJP2 gene c.3160A>G (p.T1054A), and the UGT1A1 gene c.211G>A (p.G71R). This indicates that the patient also carries two mutated genes: a TJP2 gene mutation associated with progressive familial intrahepatic cholestasis type 4 and a UDP-glucuronosyltransferase (UGTs) gene mutation associated with Crigler-Najjar syndrome.

Upon admission, the patient presented with recurrent abdominal pain and abdominal distension without obvious triggers. The pain was mainly located in the upper abdomen below the xiphoid process, presenting as intermittent colic. After receiving analgesic treatment, the upper abdominal pain gradually subsided, with abdominal distension becoming the main symptom, accompanied by such discomfort as nausea, vomiting, and reduced anal exhaust. Abdominal contrast-enhanced CT suggested incomplete intestinal obstruction. The patient was advised to fast and underwent gastrointestinal decompression, enema, acid suppression, gastric mucosal protection, anti-infection, liver protection, enzyme reduction, jaundice relief, nutritional support and fluid replacement therapy. After fasting, the patient had reduced bowel movements, weakened bowel sounds, recurrent abdominal pain, and increased ascites. Fluid accumulation was observed in the liver and kidney fossa, with a maximum depth of 67 mm in the lower right abdomen, and multiple intestinal tubes were visible. The drainage fluid from the abdominal cavity was turbid and reddish, results are mentioned in table 3.

### Ascitic Fluid Analysis Report

**Table 3:** 

Parameter	Patient Value	Normal Range (Chinese reference)
Nucleated Cell Count	120 × 10⁶/L	< 500 × 10⁶/L (PMN < 250)
Red Blood Cell Count	12 × 10⁶/L	< 10 × 10⁶/L
Mucus Qualitative Test	Weakly positive	Negative
Neutrophil Proportion	0.31	< 25%
Lymphocyte Proportion	0.69	< 50%
Total Protein (g/L)	22.1	< 25 g/L (transudate) or >25 g/L (exudate)
Lactate Dehydrogenase (LDH)	47.1	< 200 U/L
Albumin (g/L)	14.3	Used with serum albumin (SAAG)
Adenosine Deaminase (ADA)	1.4	< 10 U/L
Tumor Markers	NIL	Negative/within serum range
Ascites Drainage Volume/day	600–1000 mL	Normally absent
Gastric Tube Drainage/day	100–200 mL (yellow-green)	Normally absent

The patient's urine was brown under normal light while wine-red under sunlight. Ten days later, the patient experienced severe abdominal pain again. An urgent abdominal CT scan revealed suspected small bowel torsion with obstruction. After multidisciplinary consultation, conservative treatment was recommended. During the course of the disease, the total bilirubin level peaked at 442 μmol/L, with direct bilirubin at 330 μmol/L and indirect bilirubin at 112 μmol/L. The patient experienced recurrent symptoms of intestinal obstruction and reduced anal flatulence. After more than a month of treatment, the patient's symptoms improved and they were discharged, all the events and symptoms are mentioned below in figure 4.

**Fig 5:**
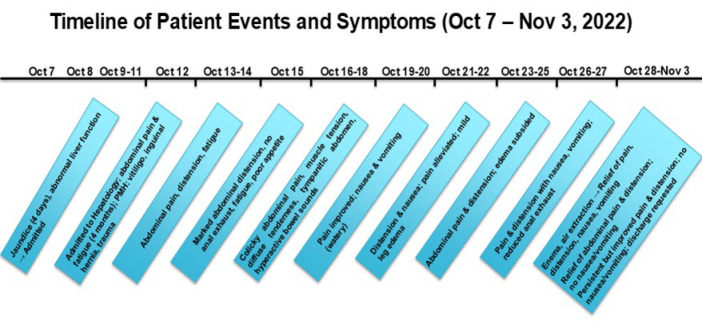
TimeLine of the pre-treatment and post-treatment events.

## Discussion

Hepatic porphyria comprises a group of rare, inherited metabolic disorders characterized by partial deficiencies in specific enzymes primarily affecting hepatic function (10). These disorders typically present with acute, severe, and diffuse abdominal pain that is often out of proportion to physical examination findings and can mimic a surgical abdomen in the absence of peritoneal signs (11). Associated gastrointestinal features may include abdominal distension, secondary to autonomic neuropathy or paralytic ileus, as well as constipation, nausea, and hypoactive bowel sounds (12, 13). Systemic manifestations commonly include autonomic dysfunction (e.g., tachycardia, hypertension), hyponatremia due to syndrome of inappropriate antidiuretic hormone secretion (SIADH), motor neuropathies, proximal muscle weakness, and central nervous system involvement such as seizures, confusion, hallucinations, and psychiatric disturbances (e.g., anxiety, depression) (14, 15). The diagnostic challenge arises from the protean and nonspecific nature of symptoms, frequently resulting in misdiagnosis or delayed diagnosis. Biochemical confirmation during an acute attack requires quantitative measurement of urinary porphobilinogen (PBG) and δ-aminolevulinic acid (ALA), both of which are markedly elevated. Genetic testing for pathogenic variants in HMBS (AIP), CPOX (HCP), PPOX (VP), or ALAD (ALADP) provides definitive diagnosis and allows for familial screening. Recent advances have significantly transformed the therapeutic landscape of hepatic porphyrias, particularly acute hepatic porphyrias (AHP). The cornerstone of acute attack management remains intravenous hemin, which downregulates hepatic ALA synthase 1 (ALAS1), reducing the accumulation of neurotoxic precursors δ-aminolevulinic acid (ALA) and porphobilinogen (PBG). Givosiran has demonstrated significant efficacy in reducing attack frequency and improving quality of life, though high cost and limited accessibility remain barriers in many healthcare settings (16, 17). Supportive therapy includes aggressive symptom control with opioids for pain, antiemetics, fluid and electrolyte correction—particularly hyponatremia—and elimination of precipitating factors such as porphyrinogenic drugs, fasting, caloric restriction, infections, and hormonal fluctuations (e.g., luteal phase of the menstrual cycle) (5, 8, 18). For patients with recurrent attacks or chronic symptoms, prophylactic treatment with givosiran, a liver-targeted RNA interference therapeutic that selectively inhibits ALAS1 mRNA, has demonstrated efficacy in reducing attack frequency and improving quality of life (19). Long-term care also necessitates comprehensive strategies for neuropathic pain management, psychological support, and judicious opioid use to minimize the risk of tolerance and dependency (20). Early recognition and a high index of clinical suspicion, coupled with a multidisciplinary management approach, are essential to mitigating morbidity and preventing long-term sequelae in patients with hepatic porphyrias presenting with recurrent abdominal pain and distension.

Acute intermittent porphyria (AIP) is a dominant genetic disorder that affects approximately 5 to 10 individuals per 100,000 population. It is difficult to diagnose in children before puberty (21). While most of AIP patients have mutations in the w198x, r173w, and r167w genes, these mutations are not necessarily associated with the disease (22), and only some of mutation carriers exhibit symptoms (23). However, our patient also carries the c.587G>T (p.C196F), TJP2 gene c.3160A>G (p.T1054A), and UGT1A1 gene c.211G>A (p.G71R) mutations. Perhaps this is the reason for his severe illness and poor response to treatment. There is no significant racial and gender difference in the onset of AIP, but female patients are more likely to exhibit symptoms (24). Both acute and induced symptoms are caused by genetic defects in several enzymes in the heme synthesis pathway, which lead to recurrent neurovisceral symptoms (25). These symptoms typically manifest as abdominal pain, but may also include neurological disease, muscle weakness, and central nervous system dysfunction (12). 

Hepatic porphyria is a porphyrin metabolism disorder caused by congenital enzyme defects in the liver. The intermediate metabolite uroporphyrin is insoluble in water and can only be excreted through the bile duct via bile (26). When there is an excess, it can lead to bile stasis at various levels of the liver cells and bile ducts, and even develop into cirrhosis, liver failure, and portal hypertension (27). The main clinical manifestations are skin changes after repeated exposure to sunlight, intractable abdominal pain, intermittent intestinal obstruction, and hyperbilirubinemia (28). In our case study, the diagnosis of hepatic porphyria was made by combining pathological and genetic testing. As described earlier in the results, the patient's liver tissue showed typical porphyrin lesions: hepatocyte, more small bile ducts and capillaries dilated and precipitated, visible brown Maltese cross-shaped inclusions under polarized light microscopy, Masson and Picrosirius Red stains showing Abasi fibers, bridging fibrosis, and positive PAS. The bile stasis in the patient's liver cells and bile ducts was very severe, causing the patient's jaundice to gradually worsen. Due to the lack of specific drugs, the patient repeatedly experienced intestinal obstruction, intractable nausea, vomiting, and poor nutritional status, resulting in poor treatment outcomes.

Treatment for hepatic porphyria is based on removing the cause and supporting symptomatic relief. Hematin supplementation therapy is effective for most patients (29). In 2019, the US FDA approved Givlaari (Givosiran) for the treatment of acute hepatic porphyria in adults (17), but most hospitals lacks this drug. Liver transplantation may be effective in the short term (30), but it does not change the genetic defect. Avoiding sunlight exposure and using zinc oxide or titanium dioxide ointment topically on the skin can provide some protection (31). Beta-carotene and alpha-melanocyte-stimulating hormone can help improve tolerance to sunlight. Gene therapy, such as antisense oligonucleotides, which prevent abnormal splicing of FECH mRNA and increase FECH enzyme activity, is a new direction for clinical research (32). In our patient, recurrent abdominal pain, abdominal distension, and progressive jaundice mirrored the nonspecific but severe presentations of acute hepatic porphyrias often reported in the literature. While supportive measures were applied, the lack of access to targeted therapies such as intravenous hemin or givosiran—shown to reduce attack frequency and neurotoxic precursor accumulation—likely contributed to recurrent episodes and poor outcomes. Recent reports also highlight the role of advanced interventions, including liver transplantation, in refractory cases, underscoring the importance of early recognition and genetic confirmation to improve prognosis. In addition to targeted therapy, the patient received extensive symptomatic and supportive management. The treatment regimen included multiple therapeutic measures. To promote bowel movements, lactulose, Liuwei Anxiao Capsules, and concentrated sodium plus glycerin enemas were administered. Simethicone was used to expel intestinal gas, supplemented by manual air extraction and anal tube decompression. Mosapride citrate was prescribed to enhance gastric motility, while lansoprazole provided acid suppression and teprenone offered gastric mucosal protection. Octreotide was employed to inhibit pancreatic enzyme secretion. Magnesium sulfate and phloroglucinol were given for spasmolysis and pain relief, and metoclopramide was administered for antiemesis. Probiotics were used to regulate the intestinal flora. Nutritional support included placement of a jejunal feeding tube to strengthen enteral nutrition, along with fat emulsion, amino acids, and glucose supplementation. Additional supportive measures consisted of electrolyte replacement, fluid therapy, acupuncture, and traditional Chinese medicine. These measures alleviated intestinal obstruction, restored anal exhaust, and allowed gradual resumption of oral feeding before discharge.

Upon discharge, the patient was instructed to maintain a balanced diet with adequate calorie intake, prioritize carbohydrate-rich foods such as rice and noodles, and increase the intake of iron-rich foods including lean meat, animal liver, and beans. Lifestyle recommendations included avoiding alcohol and direct sunlight, ensuring sufficient sleep and rest, and minimizing overwork and psychological stress. Regular hospital follow-up was advised to monitor blood routine, liver and kidney function, and urinary porphyrins. During follow-up, the patient continued to experience intermittent abdominal distension, particularly aggravated by oral intake, and therefore opted for nutritional support through a jejunal drainage tube. Despite these interventions, the patient eventually discontinued further treatment and passed away one month after discharge.

## Conclusion

This case highlights the diagnostic and therapeutic challenges of hepatic porphyria presenting with persistent abdominal pain, abdominal distension, and progressive jaundice. Despite comprehensive supportive therapy, the absence of access to targeted treatments such as intravenous hemin or givosiran led to repeated attacks and poor clinical outcomes. Pathological and genetic confirmation underscored the severity of hepatic involvement and the importance of considering porphyria in patients with unexplained abdominal symptoms and abnormal liver function tests. Early recognition, genetic testing, and timely initiation of disease-specific therapies are essential to improve prognosis, while our case further illustrates the fatal consequences of delayed diagnosis and limited treatment availability.

## List of Abbreviations

AIP - Acute Intermittent Porphyria

HCP - Hereditary Coproporphyria

VP - Variegate Porphyria

ALADP - ALA Dehydratase Deficiency Porphyria

AIP - Acute Intermittent Porphyria

FECH - Ferrochelatase

TJP2 - Tight Junction Protein 2

UGT1A1 - UDP-Glucuronosyltransferase 1A1

ALT - Alanine Aminotransferase

AST - Aspartate Aminotransferase

GGT - Gamma-Glutamyl Transferase

CRP - C-Reactive Protein

INR - International Normalized Ratio

CT - Computed Tomography

MRI - Magnetic Resonance Imaging

ECG - Electrocardiogram

CEA - Carcinoembryonic Antigen

CA 50 - Cancer Antigen 50

CA 125 - Cancer Antigen 125

LDH - Lactate Dehydrogenase

ADA - Adenosine Deaminase

hs-CRP - High-Sensitivity C-Reactive Protein

PBG - Porphobilinogen

ALA - δ-Aminolevulinic Acid

## Appendix

Supplementary table S2
